# Host combats porcine reproductive and respiratory syndrome virus infection at non-coding RNAs level

**DOI:** 10.1080/21505594.2024.2416551

**Published:** 2024-10-15

**Authors:** Zhi Qin, Weiye Liu, Zhihua Qin, Hongliang Zhang, Xuewei Huang

**Affiliations:** a College of Mechanical and Electrical Engineering, Qingdao Agricultural University, Qingdao, P.R. China; b College of Veterinary Medicine, Qingdao Agricultural University, Qingdao, P.R. China

**Keywords:** Porcine reproductive and respiratory syndrome virus, microRNAs, long non-coding RNAs, circular RNAs, viral infection

## Abstract

Porcine reproductive and respiratory syndrome virus (PRRSV) poses a significant threat to the global swine industry. The emergence of new, highly virulent strains has precipitated recurrent outbreaks worldwide, underscoring the ongoing battle between host and virus. Thus, there is an imperative to formulate a more comprehensive and effective disease control strategy. Studies have shown that host non-coding RNA (ncRNA) is an important regulator of host – virus interactions in PRRSV infection. Hence, a thorough comprehension of the roles played by ncRNAs in PRRSV infection can augment our understanding of the pathogenic mechanisms underlying PRRSV infection. This review focuses on elucidating contemporary insights into the roles of host microRNAs (miRNAs), long non-coding RNAs (lncRNAs), and circular RNAs (circRNAs) in PRRSV infection, providing both theoretical foundations and fresh perspectives for ongoing research into the mechanisms driving PRRSV infection and its pathogenesis.

## Introduction

Porcine Reproductive and Respiratory Syndrome (PRRS) is a disease characterized by respiratory tract distress in piglets, reproductive failure in sows, and high mortality, attributed to the PRRS virus (PRRSV). PRRSV, an enveloped and single-stranded RNA virus, belongs to the *Arteriviridae* family within the *Nidovirales* order [1]. The virus is categorized into two species: type 1 and type 2. The initial outbreaks of PRRS were reported almost concurrently in the United States (late 1980s, genotype 2) and Europe (early 1990s, genotype 1) [2]. Subsequently, the disease rapidly spread to other swine-raising regions worldwide, resulting in catastrophic economic consequences for the global swine industry.

Since the emergence and identification of PRRSV, the virus has continuously evolved, leading to recurrent global outbreaks. At the end of the 1990s, an atypical PRRSV strain caused high mortality and abortion in US pig herds [3]. In 2006, a novel highly pathogenic PRRSV (HP-PRRSV) was discovered in China, resulting in high mortality in sow populations [4]. Although the modified live vaccines (MLVs) have proven efficacy in controlling PRRS, the ongoing emergence of novel HP-PRRSV strains continues to pose a persistent threat to the swine industry [5–7]. PRRSV appears to continuously evolve by circumventing host immunity, and outbreaks caused by novel variants may persist indefinitely. More effective PRRSV-specific treatment or vaccines are and will be in great demand for the control of PRRS. A thorough understanding of the mechanisms underlying PRRSV–host interactions will greatly aid in the development of innovative vaccines.

The innate immune response serves as the primary defence mechanism against viral invasion [8]. Host pattern recognition receptors (PRRs) initiate innate immune signalling, enabling the host to counteract viral infection by recognizing virus-associated molecular patterns [9]. This initiation subsequently triggers adaptive immune responses. However, the invading viruses have also evolved various mechanisms to evade the host immune response [10]. Thus, an ongoing struggle for survival ensues between virus and the host. Upon PRRSV infection, the virus displays a remarkable ability to navigate the complexities of the host immune system, effectively evading both innate and adaptive antiviral responses [11,12]. To sustain viral replication and dissemination, PRRSV can impede innate immune signalling by targeting IFN-producing signalling pathway, JAK/STAT signalling pathways, interferon stimulated genes (ISGs), and antiviral restriction factors [11,12]. The inadequate innate immune responses worsen the ineffectiveness of the adaptive immune response to PRRSV, which can also be influenced by PRRSV through disrupting antigen presentation and impairing dendritic cells [11]. In addition to, PRRSV also evades host elimination by manipulating non-coding RNAs [11–14].

Non-coding RNA (ncRNA) refers to a category of RNA molecules that lack protein-coding capacity. Research has been indicated that protein-coding genes account for merely about 2% of the genome, while over 80% of the genome is transcribed into non-coding RNA [15]. Consequently, the abundance of ncRNAs play a pivotal role in diverse biological processes, encompassing cancer, cardiovascular diseases, and viral infections [16–18]. Classified by length and structure, ncRNAs include miRNAs, lncRNAs, and circRNAs [19]. Recent investigations have unveiled the critical involvement of ncRNAs in regulating the interplay between host cells and viruses [20–22]. Notably, PRRSV infection can prompt substantial alterations in the expression of host ncRNAs, some of which aberrantly expressed ncRNAs promote or inhibit viral replication by regulating the expression of antiviral genes [23,24]. These ncRNAs emerge as pivotal regulatory factors in the course of viral infection, particularly miRNAs. In this review, we comprehensively delineate the involvement of host miRNAs, lncRNAs, and circRNAs in modulating PRRSV replication, offering novel insights into comprehending the pathogenic mechanisms underlying PRRSV infection.

## The role of miRNAs in host response to PRRSV infection

**Figure 1.**
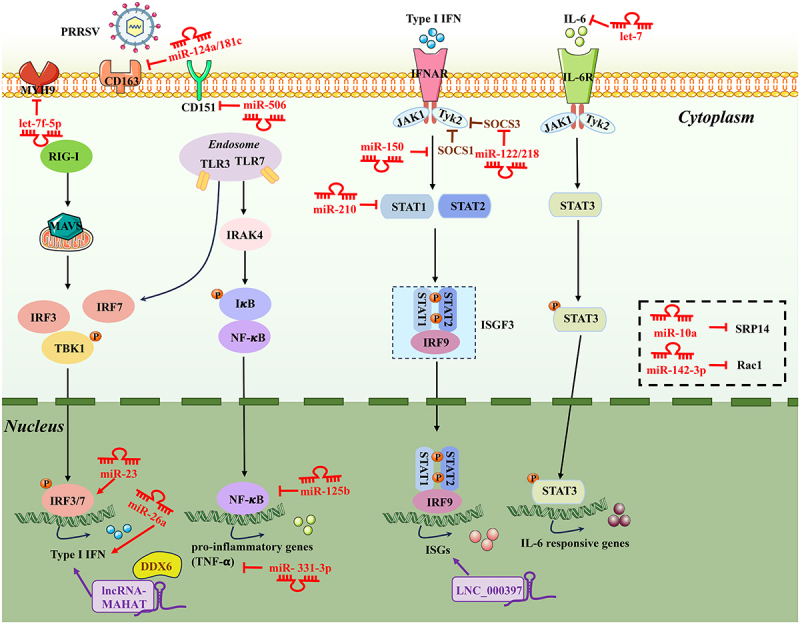
Schematic diagram of the roles of non-coding RNAs (ncRNAs) in host response to PRRSV infection. The innate immune response serves as the first line of defence against viral infections, involving the activation of multiple antiviral signalling pathways and the activation and expression of antiviral factors. PRRSV infection can induce or suppress the expression of host ncRnas, which can, in turn, indirectly inhibit viral replication and proliferation by regulating relevant antiviral factors and affecting the expression of antiviral innate immune molecules. The target gene sites involved in regulating non-coding RNAs in the innate immune response signalling pathways are concentrated in the RLR signalling pathway, nf-κB signalling pathway, and JAK-STAT signalling pathway. IL-6: interleukin 6; SOCS1/3: suppressor of cytokine signalling 3; tnf-α: necrosis factor alpha; STAT1: signal transducer and activator of transcription 1; MYH9: myosin heavy chain 9; SRP14: signal recognition particle 14.

**Figure 2.**
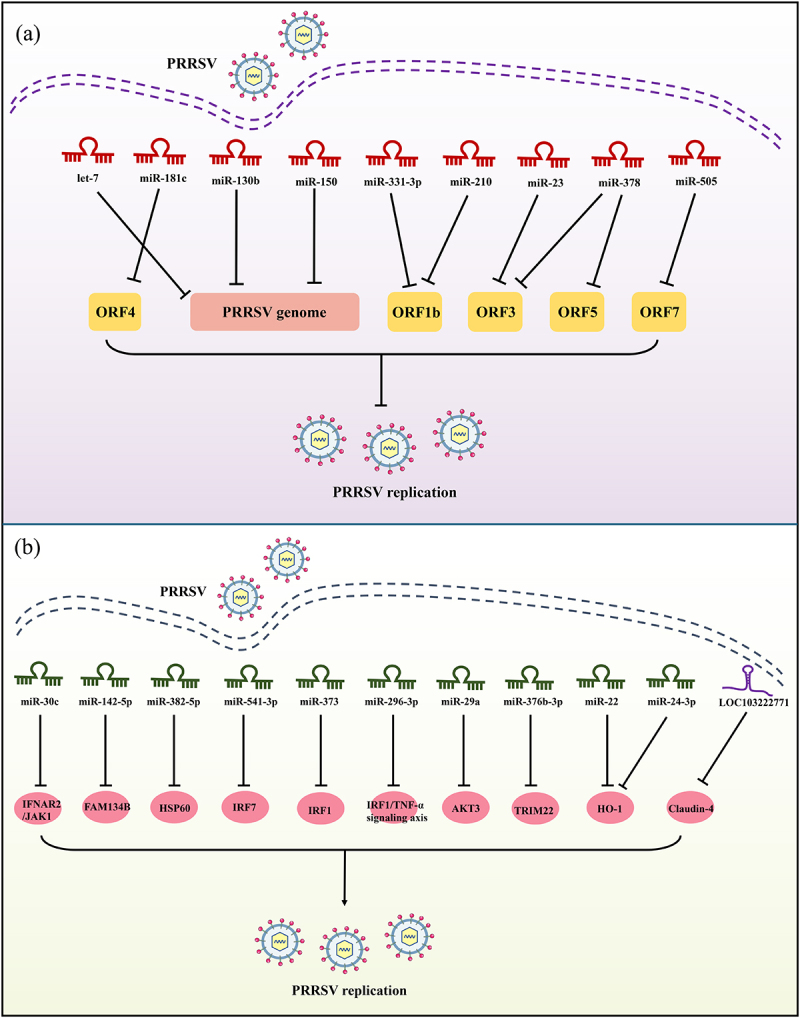
Schematic diagram of the roles of ncRNAs in host response to PRRSV infection. PRRSV infection affects the expression of ncRNAs in host cells. (a) These miRNAs inhibit PRRSV replication by directly targeting the viral genome or viral proteins; (b) these ncRNAs promote PRRSV replication by targeting antiviral host factors or the immune signalling pathways. JAK1: janus kinase 1; IFNAR2: interferon alpha and beta receptor subunit 2; HSP60: heat shock protein 60; IRF1/7: interferon regulatory factor 1/7; TRIM22: tripartite motif-containing (TRIM) 22; HO-1: haem oxygenase-1.

miRNAs, approximately 22 nucleotides (nt) in length, are endogenous small non-coding RNAs primarily responsible for mediating the degradation or translation inhibition of target genes by binding to the 3’ or 5’ untranslated regions (UTRs) [25,26]. They play crucial roles in various biological processes, including cell proliferation, apoptosis, and RNA virus infection [27,28]. Increasing evidence suggested that miRNAs participated in host antiviral responses, either by directly targeting virus genomes to regulate their translation or by targeting antiviral factors essential for virus replications [28–30]. Research has indicated that host miRNAs affected PRRSV replication through two mechanisms: firstly, certain host miRNAs can inhibit PRRSV replication by directly targeting its genomic RNA; secondly, host miRNAs can indirectly regulate PRRSV replication by targeting key genes critical for PRRSV replication. The specific details of miRNAs targeting PRRSV genome or key genes are illustrated in Figures 1 and 2, Tables 1 and 2.

**Table 1.** Host miRNAs directly target the viral genome.

miRNA	Target	Induced expression	Virus infection time	Virus	Cell Type	References
Let-7 family	PRRSV-2 genome	Up	9 hpi	WuH3	PAMs	[24]
miR-181c	ORF4	Up	24 hpi	JXwn06	PAMs	[31]
miR-130b	PRRSV genome	/	/	vJX143	PAMs	[32]
miR-150	PRRSV genome	Up	36/48 hpi	JX317648	PPMs/PAMs	[33]
miR-331-3p	ORF1b	Up	9 hpi	WuH3	PAMs	[34]
miR-210	ORF1b	Up	9 hpi	WuH3	PAMs	[34]
miR-23	ORF3	Up	12/24/36 hpi	JXwn06/CH-1a	Marc-145	[35]
miR-378	ORF3/5	/	/	JXwn06/CH-1a	Marc-145	[35]
miR-505	ORF7	/	/	JXwn06/CH-1a	Marc-145	[35]

**Table 2.** Host miRNAs indirectly regulate PRRSV replication.

miRN	Target	Induced expression	Virus infection time	Virus	Cell Type	References
Let-7 family	IL-6	Up	9 hpi	WuH3	PAMs	[24]
miR-181c	CD163	Up	24 hpi	JXwn06	PAMs	[31]
miR-150	SOCS1	Up	36/48 hpi	JX317648	PPMs/PAMs	[33]
miR-331-3p	TNF-α	Up	9 hpi	WuH3	PAMs	[34]
miR-210	STAT1	Up	9 hpi	WuH3	PAMs	[34]
miR-23	Activating IRF3/7	Up	/	JXwn06/CH-1a	Marc-145	[35]
miR-122	SOCS3	Down	24 hpi	GSWW15	PAMs	[36]
miR-218	SOCS3	Down	24 hpi	HuN4	PAMs	[37]
let-7f-5p	MYH9	Down	/	GD-HD/CH-1a	PAMs	[38]
miR-506	CD151	/	/	WuH3	Marc-145	[39]
miR-124a	CD163	Down	12/24/36 hpi	GD-HD/CH-1a	PAMs	[40]
miR-142-3p	Rac1	Down	/	HV/VR2332	PAMs	[41]
miR-10a	SRP14	Up	/	GD-HD	PAMs/MARC-145	[42,43]
miR-125b	NF-κB signalling pathway	/	/	WuH3	PAMs/MARC-145	[44]
miR-26a	Activating the IFN-I pathway	/	/	vAPRRS	MARC-145	[45]
miR-30c	JAK1/IFNAR2	Up	12/24 hpi	HV/CH-1a	PAMs	[46]
miR-142-5p	FAM134B	Up	24 hpi	WuH3	PAMs	[47]
miR-382-5p	HSP60	Up	24 hpi	BJ-4	MARC-145	[48]
miR-541-3p	IRF7	Up	24 hpi	BJ-4	MARC-145	[49]
miR-373	IRAK1/IRAK4/NFIA/NFIB/IRF1	Up	24 hpi	BJ-4	PAMs/MARC-145	[50]
miR-296-3p	IRF1/TNF-α signalling	Down	6/12/24 hpi	SHPRRS01	PAMs/MARC-145	[51]
miR-29a	AKT3	Up	24 hpi	American type PRRSV strain	PAMs	[52]
miR-376b-3p	TRIM22	Up	48 hpi	BJ-4	MARC-145	[53]
miR-22	HO-1	/	/	SD16	MARC-145	[54]
miR-24-3p	HO-1	Up	/	SD16	MARC-145	[55]

### miRNAs inhibit PRRSV replication by directly targeting the viral genome

miRNAs play an essential role in combating PRRSV infection by directly targeting the virus genome to impede virus replication. To date, nine miRNAs have been identified to exert antiviral effects on PRRSV infection, including let-7 family, miR-181c, miR-130b, miR-150, miR-331-3p, miR-210, miR-23, miR-378, and miR-505. The miRNA let-7 family, initially discovered in the *Caenorhabditis elegans*, comprises 12 members and serves as a crucial regulatory factor, influencing various biological processes [56–58]. Interestingly, the let-7 family (let-7a/c/d/e/f/g/i) was differently expressed in PRRSV-infected PAMs [24]. Let-7 family restrained PRRSV replication by directly targeting both the 3’ UTR of PRRSV-2 genome RNA and IL-6 (Figure 1 and 2a and Tables 1 and 2). The research findings indicated that the let-7 family was regulated by NEAT1 and ARID3A/NF-κB [24]. Notably, the let-7 family significantly reduced viral loads and associated pathological changes, suggesting that the let-7 family may represent a promising therapeutic target for PRRS. Moreover, the let-7 family has garnered considerable attention from numerous researchers, who have explored its intricate roles in viral infections, immunity, and various types of cancers [57,59]. It was reported that let-7b can restrict hepatitis C virus (HCV) replication by binding to the coding sequences of NS5B and the 5′ UTR of the HCV genome [60], or by directly targeting suppressor of cytokine signalling 1 (SOCS1), a negative regulator of JAK-STAT signalling [61]. Let-7c has demonstrated the ability to restrict the proliferation of HCV and influenza virus by upregulating the expression of haem oxygenase-1 (HO-1) and by targeting the H1N1 M1 gene, respectively [62,63]. As is widely known, the emergence of SARS-CoV-2 triggered a global public health crisis. Xie et al. [64] reported that members of the let-7 family can impede SARS-CoV-2 infection by targeting the M and S protein. Additionally, Saçar Demirci et al. [65] demonstrated that let-7c significantly inhibited SARS-CoV-2 replication by targeting ORF1ab. Beyond its role in viral infection, let-7 is also recognized as a tumour suppressor and a crucial immune regulatory factor, regulating cancer stemness and the inflammatory response, respectively [59]. The aforementioned research underscores the significant promise of the let-7 family as therapeutic agents for viral diseases and cancer.

miR-181c, belonging to the miR-181 family, is encoded by porcine chromosome 2. Importantly, it is the first identified miRNA capable of inhibiting the replication of genotype 2 PRRSV by targeting open reading frame 4 (ORF4) of the PRRSV genome (Figure 2a and Table 1), and administered miR-181 mimics through intranasal inhalation to inhibit PRRSV replication in an experimental porcine model [66]. Besides, Gao et al. [31] reported that transfection of miR-181 mimics can effectively restrict PRRSV proliferation by specifically targeting the PRRSV receptor CD163 (Figure 1 and Table 2). The scavenger receptor CD163 has been identified as a fusion receptor for PRRSV, playing an indispensable role in mediating PRRSV infection [67]. Moreover, pigs with CD163 knockout exhibit resistance to PRRSV infection [68]. Another study highlighted the presence of miR-181 in various pig tissues, with target gene prediction revealing perfect complementarity with Foxp1, Ddx3×, Nfat5, and Mpp5 [69]. However, delving deeper into the mechanisms involving miR-181 and its target gene requires further exploration. Indeed, members of the miR-181 family play pivotal roles in other viral infections by regulating diverse genes associated with immune responses. For example, miR-181a was shown to inhibit apoptosis *in vitro* and promoted tumour cell growth *in vivo* during hepatitis B virus infection *via* targeting TNF receptor superfamily member 6 (Fas) [70]; Mukherjee A et al. [71] reported that exogenous expression of miR-181c inhibited hepatitis C virus replication by directly binding with HCV E1 and NS5A sequences; Importantly, ongoing efforts are focused on developing infectious disease detection systems utilizing miRNAs as biomarkers, has emerged as a promising biomarker for detecting influenza H1N1 virus infection [72]. Thus, a noteworthy aspect of this family is their potential utility as potential biomarkers for diagnosing viral infections. It is interesting that miR-181 has also been demonstrated to act as a dual-function regulatory factor (acting as a tumour suppressor or oncogene) in cancer development, participating in cell proliferation, apoptosis, and autophagy [73]. Therefore, miR-181c plays diverse roles in both viral infections and cancer.

miR-130b, encoded by porcine chromosome 14, plays multifaceted roles in proliferation, differentiation, cancer progression, and autophagy [74–76]. Upon PRRSV infection, miR-130b can exert antiviral effects by directly targeting the 5’ UTR of PRRSV genome *in vitro* (Figure 2a and Table 1) [32]. It is noteworthy that the target sequence (TTGCACTG) of miR-130b on viral genome is 100% conserved in PRRSV-2 strains such as JX143, HuN4, JXM100, indicating that the target sequence is important in PRRSV-2 replication, which may be used as a potential target for new antiviral drug development. However, this sequence is absent in PRRSV-1 strains, preventing complementarity with the seed region of miR-130b, and enabling PRRSV-1 strains to evade targeting by miR-130b [32]. Importantly, intranasal inoculation of miR-130b has been shown to confer partial protection against lethal challenge with the HP-PRRSV strain vJX143, laying an important foundation for the therapeutic potential of cellular miRNAs in PRRSV infection. Additionally, miR-130b-3p can also exert antiviral effects during IBDV infection by directly targeting the genomic A segment and SOCS5 [77]. Therefore, miR-130b appears to function as an antiviral factor.

miR-150, encoded by porcine chromosome 6, is involved in multiple regulatory functions, including virus infection and cancer development. Notably, PRRSV-induced miR-150 expression by activating the PKC/JNK/c-Jun pathway [33]. miR-150 suppressed PRRSV replication *via* two distinct mechanisms. First, it directly impeded PRRSV proliferation by targeting the PRRSV genome (Figure 2a and Table 1). Second, miR-150 indirectly hampered PRRSV proliferation by binding to the 3’ UTR of SOCS1 (Figure 1 and Table 2) [33]. Moreover, miR-150-5p also can inhibit COVID-19 infection by directly targeting the virus genome Nsp10 [78]. Similar to let-7 family, miR-181c, and miR-130b, miR-150 plays a crucial role in antiviral defence.

miR-331-3p and miR-210 are encoded by porcine chromosome 5 and chromosome 2, respectively. The expression levels of miR-331-3p and miR-210 were significantly upregulated in PAMs isolated from PRRSV-2 infected pigs [34]. miR-331-3p and miR-210 can suppress PRRSV proliferation through jointly targeting PRRSV-2 ORF1b and the 3′ UTR of TNF-α and STAT1, respectively (Figure 1 and 2a and Table 1 and 2) [34]. Of note, the non-structural proteins Nsp9/10 encoded by ORF1b are associated with heightened pathogenicity and mortality in piglets [79], and disrupting ORF1b can effectively inhibit PRRSV replication [80,81]. Moreover, intramuscular injection of the miR-331-3p expression plasmid attenuated lung injury and markedly suppressed PRRSV replication *in vivo*, offering promising prospects for PRRSV treatment using cellular miRNAs. However, there is no available report concerning the impact of miR-331-3p and miR-210 on host defence against other viruses in the present study.

It was reported that miR-23, miR-378, and miR-505 exerted significant inhibition on virus replication by directly binding to the PRRSV genome, and the target sequences of these miRNAs on the viral genome show relative conservation across PRRSV-2 strains (Figure 2a and Table 1) [35]. However, this region of PRRSV-1 strains cannot complement with the seed region of miR-505 and miR-378, thus allowing PRRSV-1 to avoid being targeted by miR-505 and miR-378. Of particular interest, miR-23 has the capability to induce the expression of type I interferons by activating the IRF3/IRF7 signalling pathway during PRRSV infection, which may hinder PRRSV replication (Figure 1 and Table 2). However, the expression patterns of miR-23, miR-378, and miR-505, as well as the specific targets of miR-23 within the IRF3/IRF7 signalling pathway, remain unclear. If the expression of miR-23, miR-378, and miR-505 is downregulated by PRRSV infection, it suggests that PRRSV may hijack specific host cell mechanisms to suppress these miRNAs, thereby promoting its own survival. Conversely, if the expression of miR-23, miR-378, and miR-505 is upregulated in response to PRRSV infection, it indicates an enhanced innate immune response by the host cells against the virus. Furthermore, more efforts will be required to determine the potential targets of miR-23 within the IRF3/IRF7 signalling pathway.

### miRNAs inhibit PRRSV replication by directly targeting host factors involved in PRRSV replication

Host miRNAs can exert antiviral effects by directly targeting PRRSV receptors and the negative regulatory of immune signalling pathways. These miRNAs include miR-122, miR-218, let-7f-5p, miR-506, miR-124a, miR-142-3p, miR-10a, miR-125b, and miR-26a.

The gene for miR-122 and miR-218 are located on porcine chromosome 1 and 8, respectively. Upon PRRSV infection, the expression levels of miR-122 and miR-218 were evidently downregulated in Marc-145 cells, and delivered the mimics of miR-122 and miR-218 can strongly inhibit PRRSV proliferation by targeting SOCS3 (Figure 1 and Table 2) [36,37], a negative regulatory of JAK-STAT signalling, resulting in enhanced the expression of IFN-β. Moreover, Collison et al. [82] reported that an upregulation of miR-122 expression in the lungs of mice infected with rhinoviruses (RV), which can significantly exacerbate RV-induced lung disease by targeting the SOCS1. miR-122, a highly conserved miRNA found across vertebrate species, is predominantly expressed in the liver. It was reported that miR-122 played a central role in liver development and differentiation, contributing to liver homoeostasis cholesterol synthesis. Consequently, alterations in intrahepatic miR-122 levels are closely associated with liver diseases, including viral hepatitis [83]. Increasing evidence suggested that miR-122 can facilitate the lifecycle of HCV *via* stabilizing the viral genome, enhancing translation, and modifying the structure of the viral genome [84]. However, miR-122 May exert varied effects on different viruses. Fan *et al.* [85] and Chen et al. [86] indicated that miR-122 can inhibit hepatitis B virus (HBV) replication *via* binding to NDRG3 and the viral sequence, respectively. Therefore, miR-122 is a crucial miRNA with dual roles in viral infections. Further investigation into the molecular mechanism underlying the function of miR-122 will be essential to understand its role in the host response to pathogenic infections. Additionally, there is currently no available research on the impact of miR-218 on host defence against other viruses.

miRNAs, including let-7f-5p, miR-506, miR-124a, and miR-181c, inhibit PRRSV replication by directly targeting its receptors or essential factors for PRRSV infection. It was reported that overexpression of let-7f-5p significantly decreased PRRSV replication by directly targeting the 3′ UTR of MYH9 (Figure 1 and Table 2) [38]. MYH9 plays a pivotal role in PRRSV infection by physically interacting with the PRRSV GP5 protein *via* its C-terminal domain, thereby rendering cells susceptible to PRRSV infection [87,88]. Likewise, transfection of miR-506 mimics evidently reduced PRRSV proliferation by directly targeting the porcine CD151 receptor [39], and both miR-124a and miR-181 observably inhibited PRRSV replication by directly targeting porcine CD163 receptor (Figure 1 and Table 2) [31,40]. Of note, CD163 and CD151 serve as crucial cellular receptor for PRRSV, playing an essential role in facilitating PRRSV entry into host cells [67,89]. Similar to miR-122 and miR-218, the expression levels of let-7f-5p and miR-124a were downregulated during PRRSV infection, suggesting that let-7f-5p and miR-124a may hardly exert an antiviral activity. Additionally, accumulating evidence suggested that miR-124 was aberrantly expressed in tumour development and progression [90–94]. Similarly, let-7f-5p was also a multifunctional miRNA involved in the angiogenesis, differentiation, and pulmonary fibrosis *via* the DUSP1/Erk1/2, STAT3, and PI3K/AKT/COX2 signalling pathway [95–97]. Thus, both miR-124 and let-7f-5p exert multifaceted roles in various biological processes. However, the expression levels of miR-506 during PRRSV infection and its role in other viral infections have not been reported to date.

Host miRNAs inhibit PRRSV replication *via* other antiviral pathways. The gene transcribing miR-142-3p, miR-10a, miR-125b, and miR-26a are located on porcine chromosome 12, 12, 9, and 6, respectively. Yao et al. [41] indicated that host miR-142-3p targeted the 3” UTR of Rac1 to inhibit PRRSV replication (Figure 1 and Table 2). Rac1, a member of Rho GTPases family, plays a critical role in PRRSV entry. PRRSV infects host cells through the Rac1/Cdc42-Pak1 signaling pathway [98,99]. Moreover, the observably upregulated miR-10a during PRRSV infection inhibited PRRSV replication *via* targeting the 3′UTR of SRP14 (Figure 1 and Table 2), which can promote PRRSV replication by interacting with PRRSV Nsp2 [42,43]. Further research has identified IRF8 as a negative regulator of miR-10a. PRRSV infection decreased IRF8 expression, leading to miR-10a upregulation and thereby exerting an anti-PRRSV role [42]. Besides, both miR-125b and miR-26a have been reported to impede PRRSV proliferation by negatively regulating the NF-κB pathway and activating the IFN-I pathway, respectively (Figure 1 and Table 2) [44,100]. But the specific targets for miR-125b and miR-26a within these signaling pathways remain unidentified. Intriguingly, in addition to PRRSV, miR-10a-5p can exert an antiviral effect during porcine hemagglutinating encephalomyelitis virus (PHEV) by downregulating the Syndecan 1, a cell surface proteoglycan associated with host defense mechanisms [101]. Nevertheless, shrimp miR-10a increased white spot syndrome virus (WSSV) replication by targeting the 5” UTR of viral genes [102]. The findings imply that miR-10a may play a dual role in viral infections, serving as a double-edged sword by exhibiting both antiviral effects and promoting viral replication. However, the roles of miR-142-3p, miR-26a and miR-125b in viral infections remain relatively unexplored.

### miRNAs promote PRRSV replication

During the interaction between PRRSV and the host, host miRNAs can directly or indirectly inhibit PRRSV replication. However, numerous studies have indicated that certain host miRNAs can also negatively regulate antiviral signalling pathways, thereby reducing the expression of antiviral factors and promoting PRRSV replication. To date, 10 porcine miRNAs have been identified to facilitate PRRSV proliferation, including miR-30c, miR-142-5p, miR-382-5p, miR-541-3p, miR-373, miR-296-3p, miR-29a, miR-376b-3p, miR-22, and miR-24-3p.

Host miRNAs promote PRRSV replication by negatively regulating type I interferon (IFN-I) signalling pathway. The genes for miR-30c and miR-142-5p, situated on porcine chromosome 1 and 12, respectively. It was reported that the expression of miR-30c was markedly upregulated in PRRSV infected host cells, and overexpression of miR-30c impaired the type I interferon signalling by targeting the 3’ UTR of JAK1 (a tyrosine kinase protein) and IFNAR2 (an interferon receptor), promoting PRRSV replication (Figure 2b and Table 2) [46,103]. Importantly, *in vitro* experiments have shown that miR-30c expression was elevated in the lungs and alveolar macrophages of PRRSV-infected pigs, correlating positively with viral load [103]. Beyond PRRSV, fowl adenovirus serotype 4 (FAdV-4) can utilize gga-miR-30c-5p to target myeloid cell leukaemia-1, thereby promoting viral replication [104].

However, it was reported that gga-miR-30c-5p can also inhibit the proliferation of avian reovirus (ARV) and porcine epidemic diarrhoea virus (PEDV) by targeting autophagy related 5 and SOCS1, respectively [105,106]. This underscores the dual role of the miR-30c family in host responses to viral infections, functioning either as a proviral or antiviral factor. Guan et al. [47] reported a significant upregulation of miR-142-5p in PRRSV-infected PAMs and demonstrated that miR-142-5p facilitated PRRSV replication by directly targeting FAM134B (Figure 2b and Table 2). Notably, PRRSV replication occurs within the endoplasmic reticulum (ER), where ER phagocytosis mediated by FAM134B facilitates the activation of the IFN-I signalling pathway. Although the role of miR-142-5p in other viral infections remains unclear, its immunomodulatory effects have been confirmed in various cancers, offering valuable insights into tumour evasion of immune responses. miR-382-5p, a newly identified antiviral-associated miRNA, was upregulated during PRRSV infection. It was reported that miR-382-5p can inhibit the production of type I interferons by targeting the heat shock protein 60 (HSP60), which is a recently identified antiviral protein against PRRSV replication, thus promoting the replication of PRRSV in MARC-145 cells (Figure 2b and Table 2) [48]. Further studies revealed that HSP60 mediated the production of type I interferons by interacting with the mitochondrial antiviral signalling protein (MAVS), ultimately suppressing PRRSV replication. However, there is no report available regarding the effect of miR-382-5p on other viral infections.

Host miRNAs promote PRRSV replication by negatively regulating IRF-mediated innate immune response signalling. As a crucial interferon regulatory factor in the IFN-I signalling pathway, IRF7 can suppress the early replication of PRRSV [107]. PRRSV-2 infection induced the upregulation of miR-541-3p, which facilitated PRRSV-2 replication by directly targeting IRF7 (Figure 2b and Table 2) [49]. More and more evidence indicated that miR-541-3p acted as a tumour suppressor in the development and progression of various cancers. For instance, miR-541-3p inhibited the proliferation and metastasis of non-small cell lung cancer, hepatocellular carcinoma, and prostate cancer by targeting TGIF2, TMPRSS4, and HSP27, respectively [108–110]. Likewise, PRRSV promoted the expression of miR-373 by upregulating specificity protein 1 (Sp1) [50]. miR-373 can target interleukin-1 receptor-associated kinase 1/4 (IRAK1/4), nuclear factor IA/B (NFIA/B), and IRF1, which can reduce the production of IFN-β, thereby inhibiting PRRSV replication (Figure 2b and Table 2) [50]. Additionally, miR-373 also plays a key role in HCV infection and various cancers. It was reported that miR-373 promoted HCV replication by targeting JAK1/IRF9 and IRF5 in host cells, respectively [111,112]. Recent reports indicated aberrant expression of miR-373 in various cancers, where it can function as both an oncogene and a tumour suppressor gene [113]. These studies suggest that miR-373 may play a role in various cellular processes in both human and animal cells. Zhang et al. [114] demonstrated that transfection with miR-296-3p mimics facilitated HP-PRRSV infection by targeting IRF1, which also can regulate TNF-α expression during HP-PRRSV infection (Figure 2b and Table 2). However, unlike miR-541-3p and miR-373, miR-296-3p is significantly downregulated during PRRSV infection, indicating that the host may enhance antiviral responses by suppressing the expression of miR-296-3p. Furthermore, the role of miR-296-3p in other viral infections remains unreported.

Host miRNAs promote PRRSV replication by negatively regulating other antiviral factors. The gene encoding miR-29a is situated on porcine chromosome 18, and its expression is upregulated by PRRSV in PAMs post-infection. miR-29a facilitated early-stage PRRSV replication by targeting the 3’ UTR of AKT3 (Figure 2b and Table 2) [52]. Similarly, PRRSV components Nsp4 and Nsp11 elevated miR-376b-3p expression, which can directly target TRIM22 to disrupt its antiviral activity against PRRSV, thereby promoting PRRSV replication (Figure 2b and Table 2) [53]. TRIM22, a critical restrictive host factor, inhibits PRRSV replication by interacting with the nucleocapsid protein [115]. However, investigations into the roles of miR-29a and miR-376b-3p in other viral infections remain limited.

Moreover, host miRNAs miR-22 and miR-24-3p have been identified as facilitators of PRRSV replication by directly targeting the 3’ UTR of HO-1 (Figure 2b and Table 2) [54,55]. HO-1 is a critical cytoprotective enzyme that has been demonstrated to inhibit PRRSV replication *via* biliverdin and carbon monoxide [116,117]. Notably, miR-24-3p was markedly upregulated in PRRSV-infected Marc-145 cells. However, it remains unclear whether the expression of miR-22 in host cells was affected by PRRSV infection. If the miR-22 expression was upregulated by PRRSV infection, it was possible that some mechanism in the host cells was usurped by PRRSV to enhance the expression of miR-22 for its survival. Conversely, if the miR-22 expression was downregulated due to PRRSV infection, the innate response in host cells against PRRSV infection should be enhanced.

## The role of lncRNAs in host response to PRRSV infection

LncRNAs, typically exceeding 200 nt in length, are characterized by their lack of coding capacity, relatively low expression levels, and limited conservation [15]. They can either reside within the cell nucleus or be exported to the cytoplasm, and manifesting diverse and flexible mechanisms mediated through their specific sequences or structural motifs to interact with DNA, RNA or functional protein [118–120]. Serving as scaffolds, decoys, signals, and guides, lncRNAs play pivotal roles in regulating diverse biological processes, encompassing chromatin remodelling, transcription, translation, and RNA stability [121]. Recent studies have increasingly highlighted the indispensable roles of lncRNAs in orchestrating host antiviral responses [122–125]. The broad spectrum of activities and diverse regulatory mechanisms of lncRNAs show that lncRNAs are crucial regulatory factors in host immunity during viral infection.

### Numerous lncRNAs are identified in PRRSV infected cells

RNA sequencing (RNA-Seq) analyses have identified a plethora of lncRNAs with differential expression in PRRSV infected cells. In PRRSV-infected PAMs, a comprehensive analysis identified 86 differentially expressed (DE) lncRNAs, comprising 33 upregulated and 53 downregulated transcripts. Enrichment analyses indicated the potential involvement of these DE lncRNAs in immune responses and inflammatory processes. Co-expression networks analyses further illuminated that *IFI6*, *CXCL2*, and *CD163* were the target genes of XLOC-022175, XLOC-017089, and XLOC-019295, respectively, suggesting that lncRNAs may play a key role in immune responses against PRRSV infection by regulating their target genes [126].

Similarly, Wu et al. [127] identified 126 differentially expressed lncRNAs in HP-PRRSV infected PAMs. Subsequent investigations showed that ALDBSSCG0000007263/1674, XLOC_041472, and XLOC_008317 were associated with the antiviral-related genes, such as *MX1*, *ISG15*, *IFIT1*, and *RSAD2*, implying that these lncRNAs may play a crucial role in the antiviral response. Moreover, emerging evidence indicated that PRRSV infection altered the transcription profile of porcine trophoblast cells, and a total of 476 DE lncRNAs were identified during viral infection [128]. Notably, differential expression of lncRNAs was also observed in PRRSV infected lungs, bronchial lymph nodes, and tonsils. Co-expression network analyses elucidated that lncRNAs may positively regulate interferon gene, further suggesting their potential contribution to the antiviral response [129].

### lncRNAs promote/inhibit PRRSV replication by regulating host factors involved in PRRSV replication

Host lncRNAs play a crucial role in inhibiting PRRSV replication by modulating type I interferon signalling pathways. Recent reports have spotlighted the significant downregulation of a novel lncRNA, named lncRNA-MAHAT, upon PRRSV infection. Mechanistically, lncRNA-MAHAT functions by attenuating ZNF34 expression through the recruitment and binding of RNA helicase DDX6. Notably, the inhibition of ZNF34 promotes the expression of type I interferon, thereby impeding the replication of PRRSV (Figure 1 and Table 3) [130]. Additionally, lncRNAs can hinder PRRSV replication by regulating the expression of interferon-stimulated genes (ISGs). Of note, in both GSWW and VR2332 infection, a total of 101 and 239 DE lncRNAs were identified in GSWW and VR2332 infection group, respectively. Among these, lncRNA LNC_000397 displayed significant upregulation following PRRSV infection. Knockdown of LNC_000397 promoted PRRSV replication and markedly attenuated the expression of ISGs, including *ISG15*, *MX1*, and *RSAD2* (Figure 1 and Table 3) [131]. Given the absence of functional studies examining LNC_000397 overexpression in this study, it can only be tentatively suggested that LNC_000397 plays a pivotal role in PRRSV infection. Further investigations are warranted to fully elucidate the antiviral potential of LNC_000397 in PRRSV infection. Interestingly, lncRNAs can also facilitate PRRSV replication by regulating host factors crucial for PRRSV replication.

**Table 3.** Host lncRNAs regulate PRRSV replication.

lncRNA	Target gene	Induced expression	Virus infection time	Virus	Cell Type	References
lncRNA-MAHAT	ZNF34/DDX6	Down	24 hpi	BB0907	PAMs/Marc-145	[130]
LNC_000397	ISG15/MX1/RSAD2	Up	24 hpi	GSWW(2015)/VR2332	PAMs	[131]
LOC103222771	Claudin-4	Up	24 hpi	YN-1	Marc-145	[132]

Wang et al. [132] observed an upregulation of lncRNA LOC103222771 expression in PRRSV-infected cells, predominantly localized in the cytoplasm. Upon knockdown of LOC103222771, a decrease in PRRSV replication and an increase in Claudin-4 expression were observed in Marc-145 cells (Figure 2b and Table 3). Given that Claudin-4 exerts a negative regulatory effect on PRRSV infection, it suggests that LOC103222771 may promote PRRSV replication by negatively regulating CLDN4 [132]. Analogous to LNC_000397, the absence of functional studies on the overexpression of LOC103222771 in the study, and more efforts should be required to elucidate the antiviral function of LOC103222771 in PRRSV infection.

Until now, most research endeavours have been focused on performing RNA-Seq of cells or tissues infected with PRRSV, resulting in the identification of numerous differentially expressed lncRNAs. Nonetheless, there remains a paucity of reports elucidating the regulatory roles and mechanisms of lncRNAs in PRRSV infection. Hence, further endeavours will be required to elucidate the specific mechanisms governing lncRNA-virus interactions.

## The antiviral role of circRNAs in host response to PRRSV infection

CircRNAs are a class of endogenous regulatory ncRNAs distinguished by their prevalent occurrence and unique covalently closed-loop structure, which are evolutionarily conserved across eukaryotic evolution [133]. Substantial advancements have been achieved in elucidating the localization, modification, regulation, and degradation of circRNAs. They can exert pivotal roles in biological processes by serving as miRNA sponges, transcriptional regulators, and protein templates [134,135]. Recently, circRNAs have been identified as being dysregulated during viral infections, highlighting their significant roles in mediating virus–host interactions [114,136].

In a study, researchers employed an *in vivo* approach, subjecting piglets to PRRSV infection for durations of 3, 7, and 21 days. Subsequently, PAMs were isolated from pig lungs for RNA-Seq The results revealed differential expression of circRNAs at 3, 7, and 21 days post-infection (dpi), with 22, 171, and 84 circRNAs being differentially expressed, respectively. Among these, 13, 134, and 47 were upregulated, while 9, 37, and 124 were downregulated at the respective time points [23]. Through bioinformatics-driven construction of a circRNA-miRNA-mRNA network, circAFF3(L4,5,6).1 was identified as potentially regulating 32 genes, including THBS2, DDX60, and FGFR1, via interaction with ssc-miR-149. Similarly, circNBR1(14,L15).1 was found to potentially manipulate the expression of SHANK2, FOXJ1, and RHBDD2 by binding to ssc-miR-7135-3p. Additionally, circSPECC1 (2).1 exhibited potentially regulatory interactions with SCART1, NTN1, CASKIN2, and SULF2 through binding to ssc-miR-4332 [23]. These insights hint at a potential role of circRNAs in the host response to PRRSV infection. Further efforts will be warranted to delve deeper into the mechanisms underlying the altered landscape of circRNAs and their physiological or pathological significance in the host response to PRRSV infection.

Following PRRSV infection, a substantial number of ncRNAs are induced, including those that are upregulated and downregulated. Currently, only a few studies have confirmed the roles of ncRNAs in the interaction between the virus and the host. The functions of many ncRNAs during PRRSV infection remain largely unknown. Elucidating their mechanisms of interaction will provide researchers with a better understanding of the precise regulatory processes of these host ncRNAs during PRRSV infection. Furthermore, this knowledge could identify promising targets for developing antiviral strategies by highlighting key regulatory factors involved in viral infection and enhancing innate immune responses. However, it is noteworthy that the expression levels of ncRNAs vary depending on the strain of the virus and the timing of the infection. Whether these dysregulated ncRNAs can be simultaneously induced or act in concert remains an area for further investigation.

## Conclusions and future prospective

As researchers increasingly adopt RNA-Seq techniques, we gain more opportunities to refine genome annotations for various species. However, as the field transitions from traditional functional genomics, like microarrays, to advanced gene expression technologies based on transcriptome sequencing, there is a growing need to leverage these data not only to address fundamental questions about viral diseases but also to repurpose the same data to capture additional information that supports functional modelling. In the context of PRRSV infection, researchers have employed RNA-Seq technology to identify numerous dysregulated ncRNAs. Further investigation has revealed that many of these ncRNAs can inhibit PRRSV replication, offering promising targets for the development of future antiviral strategies. Despite the development of RNA-Seq techniques and expanding opportunities for the modulation of ncRNAs expression levels, significant challenges remain to be addressed. RNA-Seq brings with it inherent biases, limitations, and specific capacities that must be considered when developing bioinformatic tools for functional modelling [137]. These factors are crucial to ensure the accurate interpretation and application of RNA-Seq data, particularly in the study of PRRSV and related viral diseases.

NcRNAs are crucial mediators in the host response to pathogens, playing diverse roles that microbes have evolved to exploit. Understanding how ncRNAs function in the host response to infectious diseases is inherently valuable, as it helps to identify key genes and pathways that must be activated, enhanced, repressed, or silenced to facilitate an effective immune response. Through a thorough analysis of existing studies on the regulation of host ncRNAs involved in PRRSV replication, it has been found that host ncRNAs primarily regulate PRRSV replication both directly and indirectly. Some of these ncRNAs inhibit the replication and proliferation of the virus, while others aid the virus in evading the host immune surveillance, allowing for quicker invasion of host cells by interacting with viral genes. This insight provides a potential new strategy for developing anti-PRRSV drugs that target these critical viral genes.

Currently, an increasing number of miRNAs have been employed in the development of highly effective attenuated vaccines targeting various viruses [138–140]. Notably, the novel anti-miRNA treatment miravirsen is already in phase 2b trials, underscoring the potential for miRNA-based treatments to become a reality. Viral vaccines attenuated by incorporating miRNA target sequences are at the preclinical stage, and miRNA biomarkers for infection show promise. More importantly, administering mimics of miR-181, miR-130b, let-7, and miR-331-3p *via* intranasal delivery or intramuscular injection in piglets can inhibit viral replication and mitigate PRRSV-induced lung injury *in vivo* [24,32,34,69], indicating that miRNA-mediated gene silencing may be a viable strategy for managing PRRSV infections. Despite significant advances in understanding the interactions between miRNAs and their target genes in the context of PRRSV infection, many miRNAs relevant to PRRSV remain at the early preclinical stage. Thus, further investigations are imperative to develop innovative miRNA-based therapeutic drugs and overcome the challenges associated with current PRRS treatments.

The development of miRNA-based therapies faces several challenges. A single miRNA can target multiple mRNAs simultaneously, and one mRNA can be regulated by several different miRNAs [141]. This intricate network implies that therapeutic modulation of miRNA levels may have complex effects that vary depending on the microenvironment. The specific roles of miRNAs are further influenced by a variety of protein–protein and protein–RNA interactions within different contexts [142]. Furthermore, the uptake of miRNA mimics and inhibitors by different organs can result in unpredictable side effects [143]. Nonetheless, employing tissue-specific miRNAs to restrict vector tropism can be tailored to enhance specificity without diminishing efficacy [144].

LncRNAs and circRNAs are versatile and abundant transcripts involved in both normal biological activities and pathological conditions. They can exist in various forms and perform a wide range of functions. Currently, PRRSV infections are still highly prevalent in pig populations, and the effectiveness of vaccinations remains limited. Interestingly, we found that various lncRNAs and circRNAs may also be associated with PRRSV infection. Among these, certain lncRNAs can impact PRRSV replication by modulating the expression of antiviral genes. However, the majority of existing literature has primarily focused on bioinformatics analyses, and the investigation into the molecular mechanisms underlying the antiviral effects of circRNAs and lncRNAs in the host response to PRRSV infection is significantly limited, necessitating further efforts. Although therapies based on lncRNAs and circRNAs are still nascent and unlikely to transition to clinical applications in the near term, the multifaceted roles and ubiquitous presence of ncRNAs warrant more comprehensive exploration. Understanding their involvement in the host antiviral responses could pave the way for innovative strategies to effectively combat PRRSV infections.

This review consolidates existing data on miRNAs, lncRNAs, and circRNAs linked to PRRSV infections, offering insights that could guide the development of future therapeutic strategies.

## Availability of data

Availability of data sharing is not applicable to this article as no new data were created or analysed in this study.
